# Nux Vomica in Dyspepsia

**Published:** 1872-06

**Authors:** J. E. Nichols

**Affiliations:** Osage, Iowa


ARTICLE II.—*Nux Vomica in Dyspepsia. Small Doses of Medicine Generally.* By J. E. NICHOLS, M.D., Osage, Iowa.

I know of hardly anything that is such a source of annoyance to both patient and physician as a pain in the stomach, or deranged epigastric region. When an individual, melancholic, pale, cadaverous, and careworn, comes moping into my office with his hand embracing the right or left hypochondriac region, I at once settle myself for a long siege, and expect to be bored with a full account of innumerable trials with, and hair-breadth escapes from, other doctors. I am seldom disappointed. They have been from Dan to Beersheba; have taken medicine by the barrel, pailful and glassful; have swallowed boluses, pills and pellets; they have eaten largely, and have often starved; they have consulted Hahnemann's disciples, breathed atmosphere from small vials, and lived exclusively on Graham bread; but still the cry remains the same: "My stomach! Oh, my stomach!"

My course with such patients thus far has been very satisfactory to all concerned. I listen patiently to all they have to say in the first instance, and in the next, I make a full and very satisfactory (to the patient) examination of his organism. I begin at the stomach with my inquiries, and from there radiate to the circumference in every direction, dwelling upon the condition of the bowels, kidneys, lungs, heart, and nervous centres. To the question they invariably ask: "Well, doctor, can you cure me?" I as invariably and emphatically answer that I can. I only ask them to give me time, and they shall know how it seems to be without a stomach to nurse.

My main reliance is nux vomica; I use it in the form of fluid extract. The Professor of Principles and Practice of Medicine of my *Alma Mater*, gave us the name of a cure-all, or the superior of all patent medicines; its name is "good healthy *blood*," and I find it greatly to my credit to obtain the manufacture of this article as soon as possible in all of these old chronic cases. For the purpose of accomplishing this, I give the nux vomica in small tonic doses—in fact, large doses, with an idea of bringing about a radical change in any case, I find absolutely injudicious. I believe that in the treatment of chronic affections, as well as in many acute diseases, this mistake of overdosing is more frequently made than any other. If there is little or no trouble beyond the stomach, I give

R. Fl. Ext. Nux Vom., - - dr. iij.
Alcohol, - - - - - dr. vj.
M. Sig. Take 5 drops in a little water before each meal.

I tell them to take nothing that does not agree with them. If the five drops produce pain, and it often does, or they so imagine, take but one at first, then two, and gradually increase the dose to five drops, which is the extent of the dose at any time. In no case have I ever prescribed this remedy without improvement in from three to ten days. I keep them upon it for two or three months, resulting in a perfect cure.

If there is some irregularity of the bowels, with the pain of epigastrium extending around to the right hypochondrium, I combine the mandrake as follows:

R. Fl. Ext. Nux Vom.,
Fl. Ext. Mandrake,
Alcohol, - - - - - *aa* dr. iij.
M. Sig. As the other.

If there is much complaint of wakefulness at night, horrid dreams, etc., I give, in addition to the foregoing:

R. Iodide Potassium, - - - dr. ss.
Chlorate Potassium,
Carbonate Potassium, - - - *aa* dr. j.
M. Ft. Pulv. Div. in Chart. xx.
Sig. Take one in half a glass of warm milk each night at bedtime.

If, as is often the case, great portions of the day witnessed pain in the head, throbbing in the temples, I give bromide of potassium and syrup for a morning potion, to be taken as soon as awake. Sometimes I combine gelseminum with my *magnum bonum*, thus:

R. Fl. Ext. Nux Vom.,
Fl. Ext. Mandrake,
Fl. Ext. Gelseminum, - - - *aa* dr. iij.
M. Sig. As the others.

Again, I use the cannabis indicus in place of the bromide or gelseminum in cases of nervous irritability.

In cases of troublesome nausea, heartburn, acrid or fetid eructations, after eating, I use phosphate of lime rubbed up with loaf sugar, and allow them to take small quantities of it often; or, in place of this:

R. Syr. Auranti, - - - oz. vj.
Arom. Sul. Acid, - - - dr. ij.
M. Sig. Take a teaspoonful an hour after each meal, or whenever the eructations are troublesome.

Thus, with nux vomica for my central figure, I rally around it with one or more of the others as above enumerated, always telling my patient to never take the second dose if the first don't fit, or if it does not make the stomach feel better. In over fifty cases treated the last two years, not one has failed to recover, and, so far as I know, all are now strong and hearty. They at first are very anxious to know what they may eat, and this is no trivial matter. I invariably urge the use of beefsteak; if distasteful to them, begin with a small piece, as rare cooked as may be, and continue to try until they have a relish for it. Outside of this they are to eat *whatever the appetite demands*, unless they find it disagrees with them, and to eschew all such food as distresses them shortly after taking it, with the promise that in a few weeks they may eat whatever they desire without harm. I generally find it difficult to get them to eat enough. Had I not found this treatment equally good in Southern Illinois before coming here, I should have attributed some of its success to the high latitude of this region, for I have had patients that have visited our large cities and consulted eminent physicans without relief, whose difficulties have readily yielded to this small-dose treatment here.

I want to enter here my most solemn protest against all this extensive dosing with all manner of drugs compounded with poor whisky, and poured down dyspeptic patients by the glassful. When a stomach becomes so weakened and irritable that it will bear scarcely any bland and palatable food, I cannot understand how all this stimulating, exciting and nauseating slop can prove beneficial. On the other hand, I can understand that the smallest possible potion of some bitter tonic, gradually increased and always in a condensed form, will at once give tone to this highly useful and sensitive organ—the stomach. Not only do I find it profitable in these old chronic difficulties to lessen the dose, but in nearly all cases to which I am now called, I give much smaller doses than formerly, with marked result for the better. If at any time there is any doubt in regard to quantity, I give the smaller dose the benefit of the doubt.

In our vicinity are two or three doctors of the *old fashioned* stamp, who dispense blue mass, calomel and squills as freely as was done twenty-five years ago. Several times I have been called to cases that they have incidentally or otherwise seen, and have declared regarding them that they must have a "run of the fever," when the result has proved that a little sane treatment has brought them up in a few days. I give them credit for good judgment in prognosis, if *they* had treated the cases, for I verily believe that if these cases had been dosed extensively, it would have been a three weeks run to either the state of convalescence or — the grave.

